# The Effects of *Lactobacillus acidophilus* on the Intestinal Smooth Muscle Contraction through PKC/MLCK/MLC Signaling Pathway in TBI Mouse Model

**DOI:** 10.1371/journal.pone.0128214

**Published:** 2015-06-01

**Authors:** Bo Sun, Chen Hu, Huan Fang, Lina Zhu, Ning Gao, Jingci Zhu

**Affiliations:** 1 School of Nursing, Third Military Medical University, Chongqing, 400038, China; 2 College of Pharmacy, Third Military Medical University, Chongqing, 400038, China; Temple University School of Medicine, UNITED STATES

## Abstract

Clinical studies have shown that probiotics influence gastrointestinal motility. However, the molecular mechanisms by which probiotic *Lactobacillus *modulates intestinal motility in traumatic brain injury (TBI) mouse model have not been explored. In the present study, we provided evidence showing that treatment of TBI mice with *Lactobacillus acidophilus* significantly improved the terminal ileum villus morphology, restored the impaired interstitial cells of Cajal (ICC) and the disrupted ICC networks after TBI, and prevented TBI-mediated inhibition of contractile activity in intestinal smooth muscle. Mechanistically, the decreased concentration of MLCK, phospho-MLC_20_ and phospho-MYPT1 and increased concentration of MLCP and PKC were observed after TBI, and these events mediated by TBI were efficiently prevented by *Lactobacillus acidophilus* application. These findings may provide a novel mechanistic basis for the application of *Lactobacillus acidophilus* in the treatment of TBI.

## Introduction

Traumatic brain injury (TBI) occurs when an external force traumatically injures the brain. TBI is a major cause of death and disability worldwide. A World Health Organization study estimated that 70–90% of patients suffer a mild TBI, a substantial number of patients suffer a severe TBI [1]. Increasing evidence shows that TBI can lead to several physiologic complications including gastrointestinal dysfunction [2]. Traumatic brain injury causes a delayed but significant decrease in intestinal contractile activity, which is associated with disturbances in food intake and inadequate nutrition [3]. Therefore, development of optimal treatment strategies to improve intestinal motility has important clinical significance.

Probiotics are defined as live microorganisms which, when administered in adequate amounts, confer a health benefit to the host [4]. *Lactobacillus* is one of the predominant bacteria in the animal’s GI tract [5–7] and is normal inhabitant of human gut [8]. *Lactobacillus* can produce lactic and acetic acids to lower the pH in the GI tract [9] and compete for the nutrients or epithelial adhesion sites with pathogens to inhibit the growth of pathogens [10]. In addition, *Lactobacillus* can be well tolerated in acid and bile salt and it have powerful capacity of colonization, adhesion, etc [11]. Clinical studies have shown that probiotics influence gastrointestinal motility. It has been shown that probiotic *Escherichia coli Nissle 1917* (EcN) supernatants appear to enhance human colonic contractility by direct stimulation of smooth muscle cells [12]. Budriesi R, et al. reported that crude extracts from probiotic strains contained in VSL#3 (e.g. *Bifidobacterium*, *Lactobacillus*, *Streptococcus*) are able to modulate intestinal motility by mediating proximal relaxation activity [13]. There is growing interest in developing probiotic *Lactobacillus* because of its beneficial effects on the health of the GI tract without any adverse effect [14]. However, the molecular mechanisms by which probiotic *Lactobacillus* modulate intestinal motility in TBI mouse model have not been explored.

Recent studies showed that contraction of smooth muscle is primarily mediated by the phosphorylation of 20-kDa myosin regulatory light chain (MLC_20_), and the degree and duration of contraction depend on the balance between the activities of MLCK and MLCP. It has been shown that protein kinase C (PKC) plays an important role in the regulation of MLCP activity and, consequently, in the contraction of smooth muscles [15,16]. PKC activation inhibits MLCP via phosphorylation of PKC-potentiated protein phosphatase inhibitory protein-17 (CPI-17), the endogenous inhibitory protein of the catalytic subunit of MLCP [17]. However, the role of PKC/MLCK/MLCP/MLC_20_ signaling pathway in the regulation of intestinal smooth muscle contractility of mice after TBI remains unclear.

In the present study, we investigated the effects of probiotic *Lactobacillus acidophilus* on the intestinal smooth muscle contraction in TBI mouse model. We found that treatment of mice with *Lactobacillus acidophilus* efficiently improved the contractile properties of intestinal smooth muscle, which were impaired by TBI. Mechanistically, we found that PKC/MLCK/MLC signaling pathway could play an important role in *Lactobacillus acidophilus*-mediated improvement of contractile properties of intestinal smooth muscle after TBI. These findings may provide a novel mechanistic basis for the application of *Lactobacillus acidophilus* in the treatment of TBI.

## Materials and Methods

### Ethics statement

The animal experiments were approved by the Laboratory Animal Welfare Ethics Committee of the Third Military Medical University and were performed according to the guideline on animal experiments. All animal surgery was performed under chloral hydrate anesthesia, and all efforts were made to minimize suffering.

### Animals

Male C57BL/6 mice (18–24g, 8 weeks old, n = 90), were purchased from the Animal Center of the Third Military Medical University, Chongqing, China. All procedures were approved by the Institutional Animal Care Committee and were in accordance with the guidelines of the National Institutes of Health on the care and use of animals.

### Reagents and Antibodies

Antibody against c-kit was purchased from eBioscience (San Diego, CA, USA). Antibody against MLC phosphatase (MLCP, Ab-07546) was purchased from Abcam (Cambridge, UK). Antibodies against MLC kinase (MLCK, Catalog number: 2095–1), phospho-MYPT1 (Thr853, Catalog number: #4563S), phospho-myosin light chain 2 (Ser19, Catalog number: #3675S), and GAPDH (14C10, Catalog number: 10494-1-AP) were purchased from Cell Signaling Technology (Danvers, MA, USA). The ELISA kits of Mouse MLCP (BPE06064m), Mouse PKC (BPE06013m) and Mouse MLCK (BPE06065m) were purchased from Shanghai hushang biological technology co., LTD (Qingpu district, Shanghai, China).

### TBI Procedure and Animal Management

Ninety C57BL/6 mice were randomly divided into three groups including control, TBI, and TBI + *Lactobacillus acidophilus* (TBI+La) groups (30 mice/group). Mice in each group were divided into three time points (1 day, 3 days and 7 days, 10 mice/treatment). The TBI procedure was conducted according to a modified Feeney’s method [18]. Mice were anesthetized with 10% chloral hydrate by intraperitoneal injection. After depilation and disinfection, the mice were moved to a stereotaxic frame. The scalp was opened by a midline incision, and a craniotomy was performed, with 3 mm rightward of the sagittalis suture and 3 mm posterior to the coronal suture, which was then enlarged to a bone window with 2 × 2 mm. The hitting device was fixed onto the stereotaxic frame, and the TBI was performed by releasing the cylinder bar weighing 18 g with a diameter of 3 mm from a height of 16 cm. A gelatin sponge was used to stop the bleeding, and the scalp was sutured until there was no longer any active bleeding. Mice in the control group (n = 30) underwent only opening the scalp and a craniotomy following suturing but without brain injury.

Mice in different group received the following treatments after TBI. Mice in control group (n = 30) and TBI group (n = 30) were fed with MRS (a bacterial growth medium and is named by its inventors: de Man, Rogosa and Sharp.) culture medium (0.5 ml each mouse per day) by gastric infusion and TBI + *Lactobacillus* group (n = 30) was gavaged with *Lactobacillus* (1×10^10^CFU/d) for 1, 3, 7 days, separately. Each mouse was gavaged one time a day and received a conventional balanced diet and water ad *libitum* at the rest time until the experimental procedure was initiated. Mice were housed in a temperature- and humidity-controlled environment with a 12-h light/dark cycle, room temperature 22–24°C. All mice were fed a common food with the following qualities: crude protein ≥ 17.9%, fat ≥ 4%, crude fiber ≤ 5%, crude ash ≤ 8%, 1.15% calcium, 0.85% phosphate, lysine ≥ 0.85%, and methionine ≥ 0.35%. The general status and the qualitative changes of the faeces were observed during the experiment. The terminal ileum segments of mice were taken on days 1, 3, 7 after TBI.

### Pathological Observation

The segments of the terminal ileum were removed, fixed in 10% formalin in 0.15 M phosphate buffer (pH 7.2), and then embedded in paraffin. After staining with hematoxylin-eosin, we observed the morphological change of the specimens in control group, TBI group, and TBI + *Lactobacillus* group respectively.

### The detection of contractile activity

Mice were fasted for 12 h but allowed to drink water before experiments. Immediately after each mouse was killed, a segment of ileum (1.5 cm from caecum) was removed and put into the physiological saline, then removed the mesentery with tweezer, and flushed with Krebs-Ringers solution. The isolated ileum muscle strips were longitudinally mounted in an organ bath. The organ bath was filled with 10 ml Krebs solution (36.5 ± 0.5°C) and bubbled continuously with 95% O_2_ and 5% CO_2_. Each muscle strip per mouse was placed under a preload of 0 g. Tension signal was input by tension energy transducer to the physiological recorder of multi-channel physiological signal acquisition processing system called RM6280C (Instrument Company of Chengdu, Chengdu, China). After the muscle strip was balanced in the chamber for 30 min, the one dimensional displacement spinner was adjusted to make the muscular tension increase to 0.75 g. After the muscle strip contracted spontaneously and the baseline on the recorder became stable, the tension, amplitude and frequency of contraction were observed and recorded for 15 min. Krebs solution in the bath was changed at least three times (5–10 min intervals) until the strips were stabilized between each experimental condition.

### Small intestinal transit test *in vivo*

Mice were used for a small intestinal transit test on days 1, 3, and 7 *in vivo*. Mice were gavage-fed with dextran blue-2000 (0.4 ml per mouse, 17-0360-01, GE Healthcare Bio-Sciences China Ltd, USA) and sacrificed 25 min later, and the entire small intestines were carefully harvested. The length of the small intestine was the distance from below the pylorus to the cecum. The length from below the pylorus to the anterior extremity of the dextran blue was also measured. Results were expressed as propulsion ratio (%) of the migration distance to the total length of the small intestine.

### Quantitative immunofluorescence

Segments of ileum (6 cm from caecum) were immediately removed and flushed with PBS. Tissue sections were fixed in acetone solution for 6–8 hours. Sections were then washed three times (each one lasting 5 min) in PBS and then washed in 0.05% Tween-20/0.01 M PBS for 5 min, and incubated in 1% BSA for 30 min. After rinsing, sections were incubated with the primary antibody for 2 h at RT in a moist chamber then incubated for 48 h at 4°C. Then, the sections were washed (three times in PBS, 5 min) and then washed one time in 0.05% Tween-20/0.01 M PBS for 5 min, and the secondary antibody was applied i.e. donkey to rat IgG (FITC) for 60–90 min at RT in a dark moist chamber. Sections were then rinsed three times in PBS, stained with DAPI (DNA-specific fluorescent probe, Sigma-Aldrich, USA) and then mounted in Mowiol mounting medium (Abcam, Cambridge, UK). Tissue sections in control group were processed as described above, but the serum of normal mouse instead the primary antibody. Images were viewed by fluorescence microscopy and analysed using the Leica confocal software. The number of interstitial cells of Cajal (ICC) was calculated by Image-Pro plus 5.1 software (Media Cybernetics, USA).

### ELISA

Ileum tissues were homogenized by mechanical disruption in RIPA buffer with a cocktail of protease inhibitors (P8340, Sigma, USA) and incubated on ice for 30 min. Lysates were centrifuged at 12000 rpm for 10 min and the protein content was stored at -20°C for further use. For detection of MLCK, MLCP and PKC, ninety-six-well microplates were incubated with antibodies against MLCP, PKC and MLCK at 4°C overnight. After four washes with PBS, the wells are saturated with BSA (200 μl, 3% in PBS, at least 2 h at 37°C). After four washes with PBS, 100 μl of sample containing MLCP, PKC or MLCK protein was deposited in the wells, and protein was allowed to bind to its specific antibody during 3 h at 37°C. After four washes with PBS, streptavidin-HRP (Beckman, USA) was added (100 μl, 1/1000 in PBS, 1% BSA, 30 min at 37°C). After four washes with PBS, 100 μl of HRP substrate are deposited and reaction was allowed during 10 min in dark (upon oxidation, TMB forms a water-soluble blue reaction product that can be measured spectrophotometrically at 650 nm). Reaction was then stopped by addition of 100 μl H_2_SO_4_ 2 M (upon acidification, the reaction product becomes yellow with an absorbance peak at 450 nm). Absorbance at 450 nm (yellow) was read by BioTek PowerWave 200 Microplate Scanning Spectrophotometer (Winooski, VT, USA).

### Immunohistochemistry staining

The slices of segments were dewaxed, soaked in ethanol, and then blocked with 3% H_2_O_2_. Nonspecific immunoreactivity was blocked with diluted rabbit serum at RT. The slices were incubated with antibodies against phospho-MLC_20_, MLCK, MLCP and phospho-MYPT1 4°C overnight. After washing with PBS, these slices were incubated with secondary antibodies at 37°C for 30 min. The slices were stained with a freshly prepared diaminobenzidine solution and then counter stained with Mayer’s hematoxylin. Between each step, the sections were washed with PBS. The negative control was obtained by substituting the primary antibodies with mouse IgG. Images were captured under microscope with a camera (Olympus, Japan) and analyzed with a microscope (Nikon 55I, Tokyo, Japan). Positive expression of phospho-MYPT1 showed a brown color. Integral optical density (IOD) of positive phospho-MYPT1 was quantified by image analysis. The integral optical density (IOD) of positive area was quantified by image analysis software (IPP6.0, IBM Corp., USA).

### Western blot

The ileum tissues were homogenized by mechanical disruption in RIPA buffer with a protease inhibitor cocktail (Thermo Fisher Scientific Inc., IL, USA) and incubated on ice for 30 min. Lysates were centrifuged at 12000 rpm for 10 min and the protein content of the supernatant was determined by using the BCA protein assay kit (Beyotime institute of biotechnology, Shanghai, China). After being diluted with loading buffer and heated to 95°C for 10 min. Depending on the molecular weight, a total of 40 μg of protein lysates derived from ileum tissue samples were loaded onto 12% SDS-PAGE gel. Membranes were blocked with 5% fat-free dry milk in 1× Tris-buffered saline (TBS) and incubated in 4°C with primary antibodies overnight. Protein bands were detected by incubating with horseradish peroxidase-conjugated antibodies and visualized with enhanced chemiluminescence reagent (Perkin Elmer, Boston, MA). Band intensities were quantified by the Quantity One 4.6.2 software (BioRad, USA).

### Statistical Analysis

The statistical analysis was performed by Statistical Package for Social Sciences (version 19.0, IBM Corp., USA). All data were presented as mean ± standard deviation (SD). Factorial design ANOVA was used to examine the main effect of treatment and time and their interaction. If the interaction was significant, one way ANOVA with Bonferroni correction at each time point was performed to examine the simple effect of *Lactobacillus acidophilus*. *P* < 0.05 was considered statistically significant.

## Results

### Effects of *Lactobacillus acidophilus* on intestinal contraction, ICC networks and numbers in TBI mouse model

**Fig 1:**
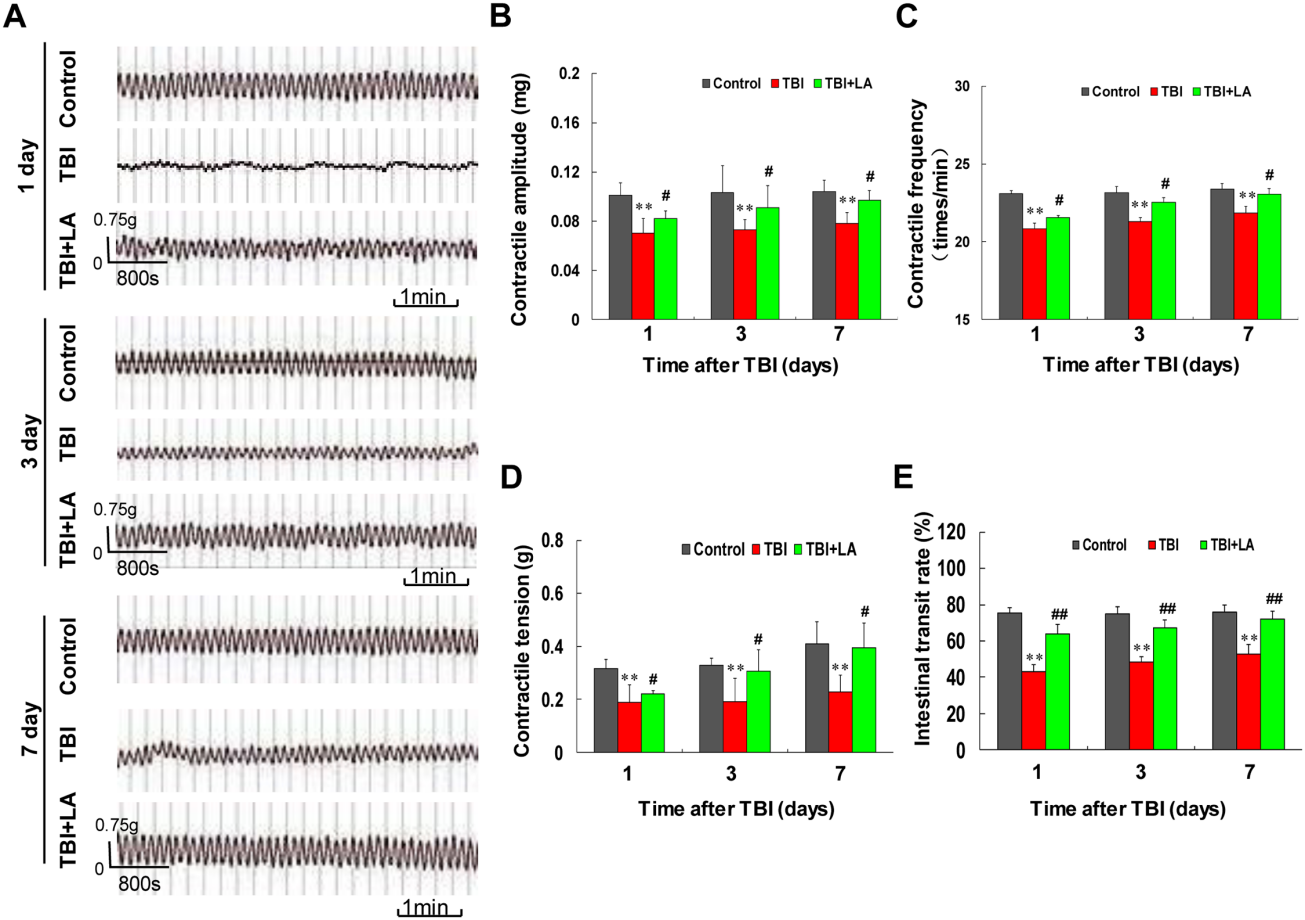
*Lactobacillus acidophilus* improved the contractile activity of intestinal smooth muscle impaired by TBI. Ninety C57BL/6 mice were randomly divided into three groups including control, TBI and TBI + *Lactobacillus acidophilus* groups. The interaction of *Lactobacillus acidophilus* and time on intestinal contractile activity was not significant. (A) The contractile activity was determined by histologic and physiologic analyses. (B) The average contractile amplitude was decreased after TBI, **P < 0.01 compared with control. *Lactobacillus acidophilus* could increase in contractile amplitude, #*P* < 0.05 compared with TBI. (C and D) The contractile frequency and tension were also decreased after TBI, **P < 0.01 compared with control. *Lactobacillus acidophilus* significantly attenuated TBI-mediated decreases of contractile frequency and tension, #*P* < 0.05 compared with TBI. (E) The intestinal transit rate was decreased after TBI, **P < 0.01 compared with control. *Lactobacillus acidophilus* significantly attenuated TBI-mediated decrease of intestinal transit rate, ##*P* < 0.01 compared with TBI.

First, we examined the effect of *Lactobacillus acidophilus* on small intestinal contractile activity in TBI mouse model. Contractile activity was measured in the small intestine from control, TBI and TBI combined with *Lactobacillus acidophilus* groups at 1, 3 and 7 days post-surgery. Table 1 shows that the interactions of *Lactobacillus acidophilus* and time on intestinal contractile amplitude, frequency and tension were not significant (*P* > 0.05). As shown in Fig 1A, TBI caused a significant decrease in intestinal contractile activity at 1 day. However, the decreased intestinal contractile activity was not more severe at 3 days and 7 days after TBI. Treatment of TBI mouse with *Lactobacillus acidophilus* resulted in significant increases in contractile activity. The contractile amplitude, frequency and tension were markedly decreased at 1 day after TBI compared with control (*P* < 0.01) and these effects were not more severe at 3 and 7 days after TBI (*P* < 0.01) (Fig 1B, 1C and 1D). Treatment of TBI mouse with *Lactobacillus acidophilus* resulted in significant increases in contractile amplitude, frequency and tension compared with TBI mice (*P* < 0.05) (Fig 1B, 1C and 1D). Meanwhile, the effects of time on intestinal contractile amplitude, frequency and tension were significant (*P* < 0.01) (Table 1).

**Table 1:** *P*-values of a two-way ANOVA for the effects of *Lactobacillus acidophilus*, time, and their interactions on the ten variables (N = 90).

Treatment	variables									
	Amplitude	Frequency	Tension	Intestinal transit	phospho-MLC_20_	MLCK	MLCP	PKC	Phospho-MYPT1	ICC numbers
*Lactobacillus acidophilus*	<0.05*	<0.05*	<0.05*	<0.01**	<0.05*	<0.01**	<0.05*	<0.05*	<0.01**	<0.05*
Time	<0.01**	<0.01**	<0.01**	0.84	<0.01**	<0.05*	0.22	<0.01**	<0.01**	<0.01**
Time×*Lactobacillus acidophilus*	0.41	0.06	0.29	0.85	0.18	0.34	0.25	0.24	0.36	0.54

Note: MLCK: myosin light chain kinase; MLCP: myosin light chain phosphatase; PKC: protein kinase C; ICC numbers: the numbers of interstitial cells of Cajal.

*indicates a significant difference (*P* < 0.05)

**indicates a highly significant difference (*P* < 0.01).

We also examined the effect of *Lactobacillus acidophilus* on the intestinal transit rate in TBI mouse model *in vivo*. Table 1 shows that the interaction of *Lactobacillus acidophilus* and time on intestinal transit was not significant (*P* = 0.85). As shown in Fig 1E, TBI significantly reduced the intestinal transit rate compared with the control group (*P* < 0.01). The reduced intestinal transit rate mediated by TBI was significantly reversed by treatment with *Lactobacillus acidophilus* (*P* < 0.01). Meanwhile, the effect of time on intestinal transit was also not significant (*P* = 0.84) (Table 1).

**Fig 2:**
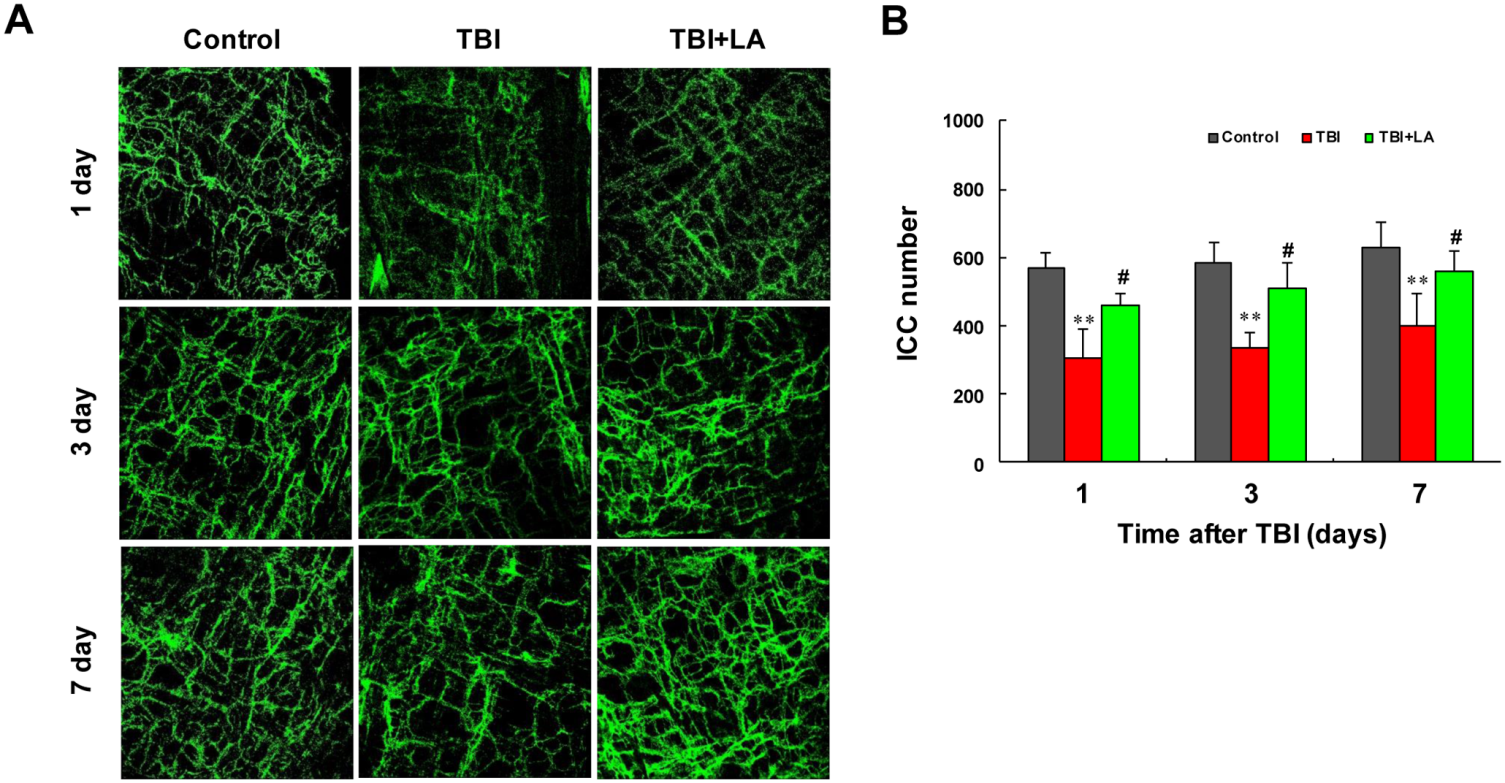
*Lactobacillus acidophilus* restored the impaired ICC networks mediated by TBI. The ICC networks and numbers were detected under fluorescence microscope (400×) by immunofluorescence in ileal tissue sections. The interaction of *Lactobacillus acidophilus* and time on ICC numbers was not significant. (A) Low-density of ICC networks was observed after TBI compared with control. *Lactobacillus acidophilus* significantly restored TBI-mediated disruption of ICC networks. (B) The reduction of ICC numbers was also observed after TBI, **P < 0.01 compared with control. *Lactobacillus acidophilus* significantly restored TBI-mediated reduction of ICC numbers, #*P* < 0.05 compared with TBI.

Since ICC play critical roles in gastrointestinal motility [19]. Maintaining the integrity of ICC networks is essential to preserve contractile activity in the gastrointestinal tract and to restore this function after TBI [20]. We next examined the effects of *Lactobacillus acidophilus* on ICC networks and numbers of the small intestine in TBI mouse model. Table 1 shows that the interaction of *Lactobacillus acidophilus* and time on ICC numbers was not significant (*P* = 0.54). As shown in Fig 2A, control mice displayed a dense network of ICC along the entire length of intestinal tract. Intact but low-density ICC networks were observed after TBI. However, treatment with *Lactobacillus acidophilus* significantly prevented TBI-mediated disruption of ICC networks. Similarly, the reduction of ICC numbers was also observed after TBI compared with control (*P* < 0.01). *Lactobacillus acidophilus* treatment significantly prevented TBI-mediated reduction of ICC numbers (*P* < 0.05) (Fig 2B). Meanwhile, the effect of time on ICC numbers was significant (*P* < 0.01) (Table 1). Taken together, these findings indicate that TBI reduced both transit rate and contractile activity of small intestine and disrupted ICC networks, and *Lactobacillus acidophilus* treatment could prevent TBI-mediated reduction of both transit rate and contractile activity and interruption of ICC networks.

### Effect of *Lactobacillus acidophilus* on small intestinal morphological alteration in TBI mouse model

**Fig 3:**
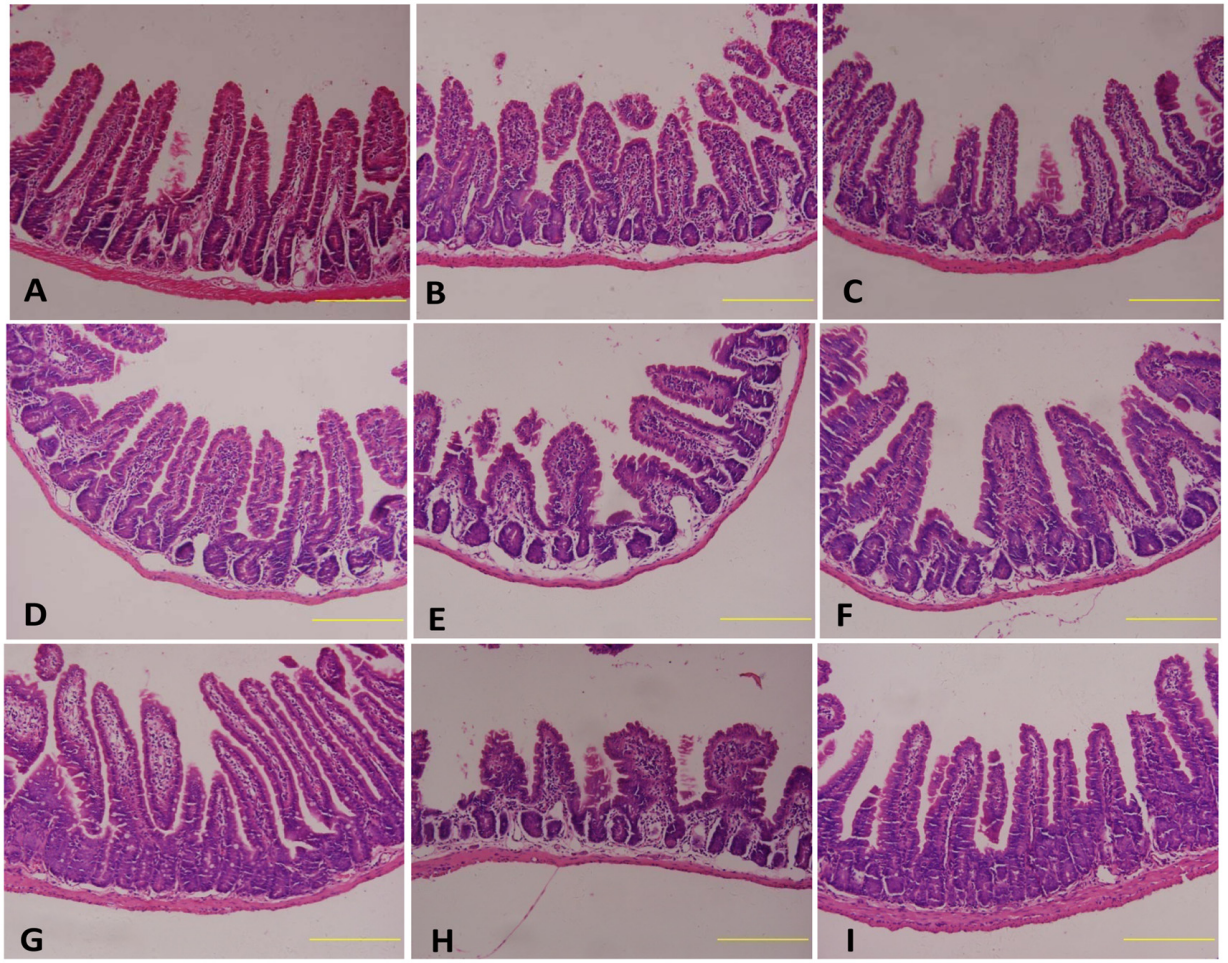
*Lactobacillus acidophilus* improved the morphology of villus. The intestinal sections from control mice presented intact structures with complete intestinal mucosae and villi (3A, 3D and 3G). TBI caused abnormal intestinal wall morphology and damaged the intestinal villi and structural integrity (3B, 3E and 3H). *Lactobacillus acidophilus* recovered intestinal mucosae and restructured villi (3C, 3F and 3I).

Next, we examined the effect of *Lactobacillus acidophilus* on small intestinal morphology in TBI mouse model. Histologic examination showed that the intestinal sections from control mice presented intact structures with complete intestinal mucosae and villi (Fig 3A, 3D and 3G). Intestinal sections from TBI mice showed a high degree of intestinal injury, with pathological charateristics including severe loss of the mucosae and damage to the intestinal villi, resulting in abnormal intestinal wall morphology and the loss of intestinal structural integrity (Fig 3B, 3E and 3H). Mice treated with *Lactobacillus acidophilus* showed intact intestine, with recovered intestinal mucosae and restructured villi (Fig 3C, 3F and 3I).

### Effect of *Lactobacillus acidophilus* on MLC_20_ phosphorylation in small intestine of TBI mouse model

**Fig 4:**
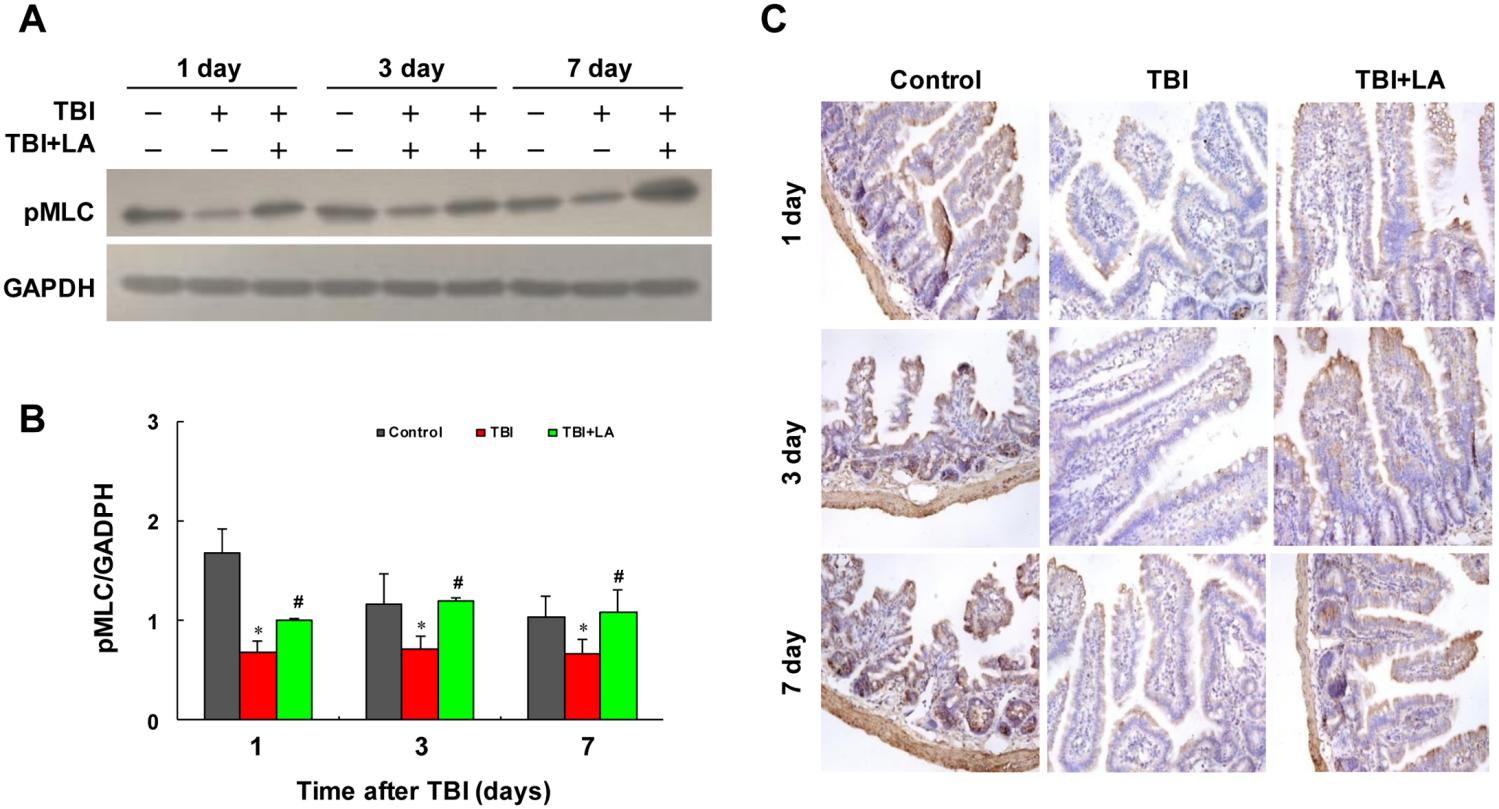
*Lactobacillus acidophilus* could increase the MLC_20_ phosphorylation. The interaction of *Lactobacillus acidophilus* and time on MLC_20_ phosphorylation was not significant. (A and B) The levels of phospho-MLC_20_ in intestinal smooth muscle were significantly decreased after TBI, *P < 0.05 compared with control. Treating with *Lactobacillus acidophilus* attenuated TBI-mediated inhibition of MLC_20_ phosphorylation, #*P* < 0.05 compared with TBI. (C) The marked decreases in immunoreactivity for phospho-MLC_20_ were observed in intestinal smooth muscle after TBI, and treatment with *Lactobacillus acidophilus* significantly attenuated TBI-mediated inhibition of MLC_20_ phosphorylation.

Recent evidence revealed that phosphorylation of MLC_20_ is a prerequisite for contraction in intestinal smooth muscle [21]. We therefore examined the effect of *Lactobacillus acidophilus* on MLC_20_ phosphorylation in intestinal smooth muscle of TBI mouse model. Table 1 shows that the interaction of *Lactobacillus acidophilus* and time on MLC_20_ phosphorylation was not significant (*P* = 0.18). Western blot analysis showed that the levels of phospho-MLC_20_ in intestinal smooth muscle were significantly decreased after TBI (*P* < 0.05) (Fig 4A and 4B). *Lactobacillus acidophilus* significantly attenuated TBI-mediated inhibition of MLC_20_ phosphorylation (*P* < 0.05) (Table 1) (Fig 4A and 4B). Meanwhile, the effect of time on MLC_20_ phosphorylation was significant (*P* < 0.01) (Table 1). Similarly, immunohistochemistry analysis showed that marked decreases in immunoreactivity for phospho-MLC_20_ were observed in intestinal smooth muscle after TBI, and treatment with *Lactobacillus acidophilus* significantly attenuated TBI-mediated inhibition of MLC_20_ phosphorylation (Fig 4C).

### Effect of *Lactobacillus acidophilus* on MLCK protein concentration in small intestine of TBI mouse model

**Fig 5:**
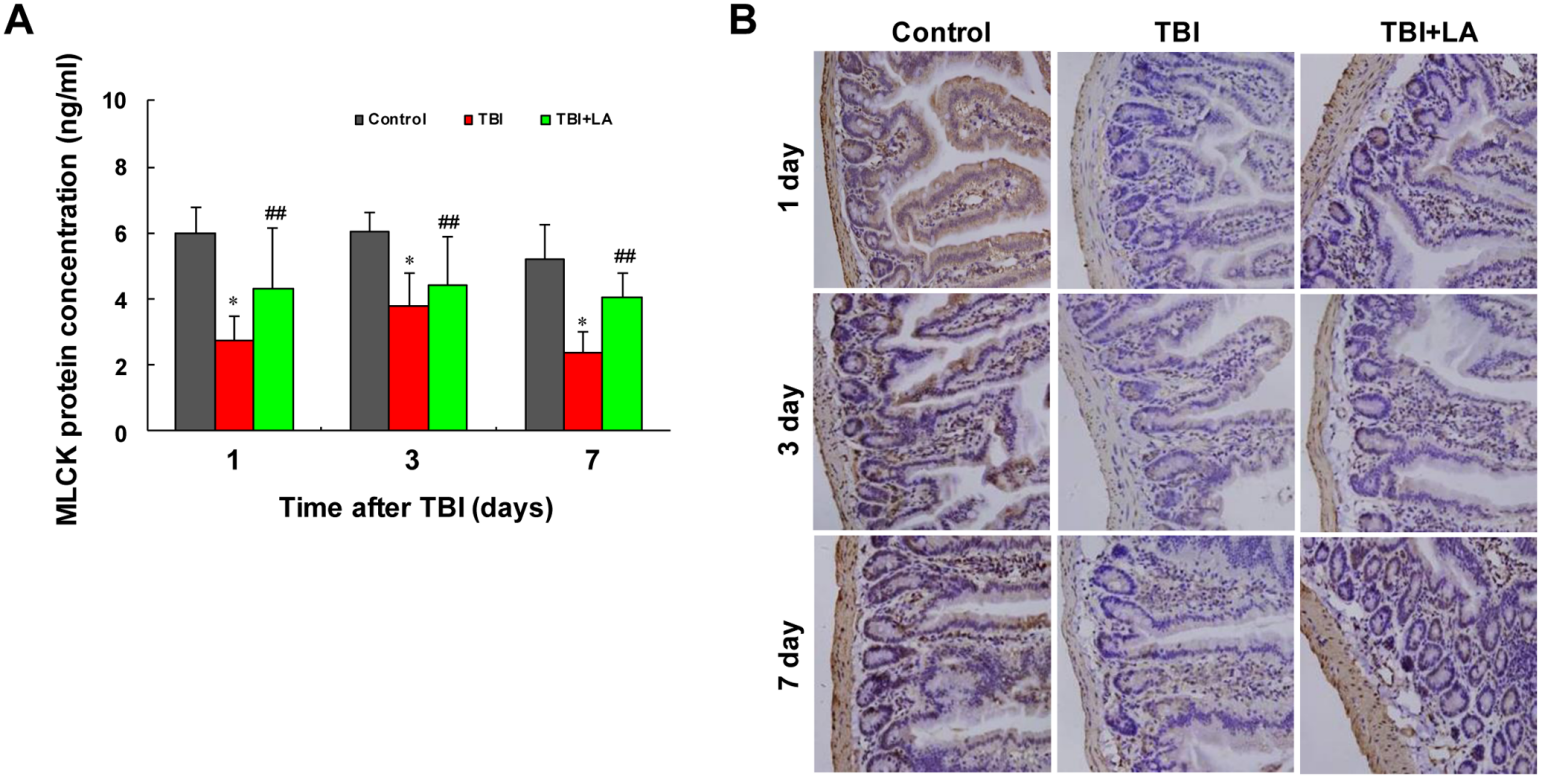
*Lactobacillus acidophilus* attenuated TBI-mediated reduction of MLCK. The interaction of *Lactobacillus acidophilus* and time on MLCK protein concentrations was not significant. (A) ELISA analysis showed that the concentrations of MLCK in intestinal smooth muscle were significantly decreased after TBI, *P < 0.05 compared with control. *Lactobacillus acidophilus* significantly attenuated TBI-mediated reduction of MLCK concentrations, ##*P* < 0.01 compared with TBI. (B) Immunohistochemistry analysis showed that marked decreases in MLCK were observed in intestinal smooth muscle after TBI, and treatment with *Lactobacillus acidophilus* significantly attenuated TBI-mediated inhibition of MLCK.

It is well known that myosin light chain kinases (MLCK) are a family of soluble protein kinases that function principally to phosphorylate the 20 kDa regulatory myosin light chain (MLC_20_) and thereby induce ATPase driven actomyosin contraction. MLCK-mediated MLC phosphorylation and actomyosin contractility are important in muscle contraction [22–24]. We next examined whether *Lactobacillus acidophilus* affects MLCK protein concentration in intestinal smooth muscle of TBI mouse model. Table 1 shows that the interaction of *Lactobacillus acidophilus* and time on MLCK protein concentration was not significant (*P* = 0.34). ELISA analysis showed that the concentrations of MLCK in intestinal smooth muscle were significantly decreased after TBI (*P* < 0.05) (Fig 5A). Treatment of TBI mice with *Lactobacillus acidophilus* significantly attenuated TBI-mediated reduction of MLCK (*P* < 0.01). Meanwhile, the effect of time on MLCK protein concentration was significant (*P* < 0.05) (Table 1). Similarly, immunohistochemistry analysis showed that marked decreases in immunoreactivity for MLCK were observed in intestinal smooth muscle after TBI, and treatment with *Lactobacillus acidophilus* significantly attenuated TBI-mediated inhibition of MLCK (Fig 5B).

### Effect of *Lactobacillus acidophilus* on MLCP protein concentration in small intestine of TBI mouse model

**Fig 6:**
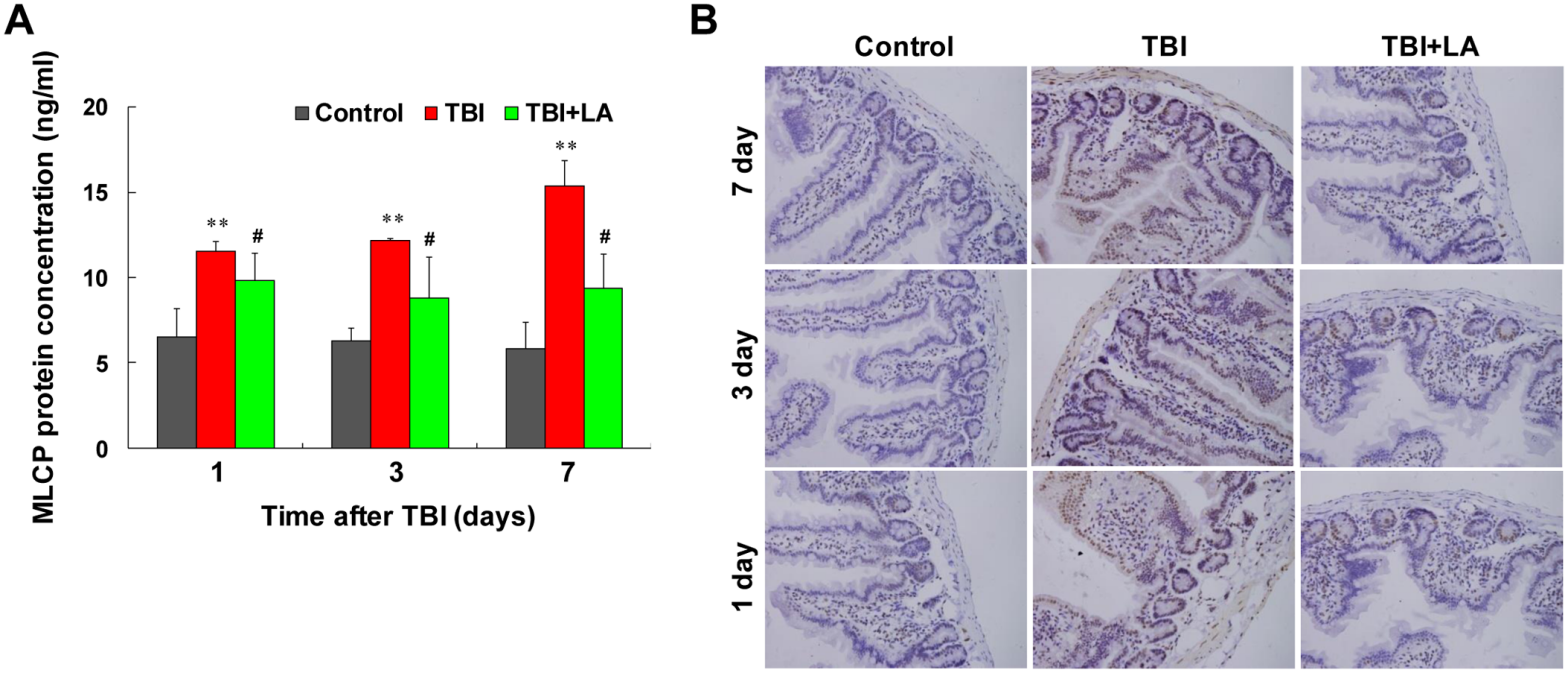
*Lactobacillus acidophilus* attenuated TBI-mediated increase of MLCP. The interaction of *Lactobacillus acidophilus* and time on MLCP protein concentrations was not significant. (A) ELISA analysis showed that the concentrations of MLCP in intestinal smooth muscle were significantly increased after TBI, **P < 0.01 compared with control. *Lactobacillus acidophilus* significantly attenuated TBI-mediated increase of MLCP, #*P* < 0.05 compared with TBI. (B) Immunohistochemistry analysis showed that marked increases in MLCP were observed in intestinal smooth muscle after TBI, and treatment with *Lactobacillus acidophilus* significantly attenuated TBI-mediated increases of MLCP.

It has been shown that the state of MLC phosphorylation involving a balance between MLCK and MLCP activities determines the contractile activity of smooth muscle [25]. MLCP is the contender, regulating the inactivation of MLC by dephosphorylation, promoting smooth muscle relaxation [26]. We therefore examined whether *Lactobacillus acidophilus* affects MLCP concentration in intestinal smooth muscle of TBI mouse model. Table 1 shows that the interaction of *Lactobacillus acidophilus* and time on MLCP protein concentration was not significant (*P* = 0.25). By using ELISA analysis, we found that the concentrations of MLCP in intestinal smooth muscle were significantly increased after TBI (*P* < 0.01) (Fig 6A). Treatment of TBI mice with *Lactobacillus acidophilus* significantly attenuated TBI-mediated induction of MLCP (*P* < 0.05) (Fig 6A). Meanwhile, the effect of time on MLCP protein concentration was also not significant (*P* = 0.22) (Table 1). Similarly, immunohistochemistry analysis showed that marked increases in immunoreactivity for MLCP were observed in intestinal smooth muscle after TBI, and treatment with *Lactobacillus acidophilus* significantly attenuated TBI-mediated induction of MLCP (Fig 6B).

### Effects of *Lactobacillus acidophilus* on MYPT1 phosphorylation and PKC protein concentration in small intestine of TBI mouse model

**Fig 7:**
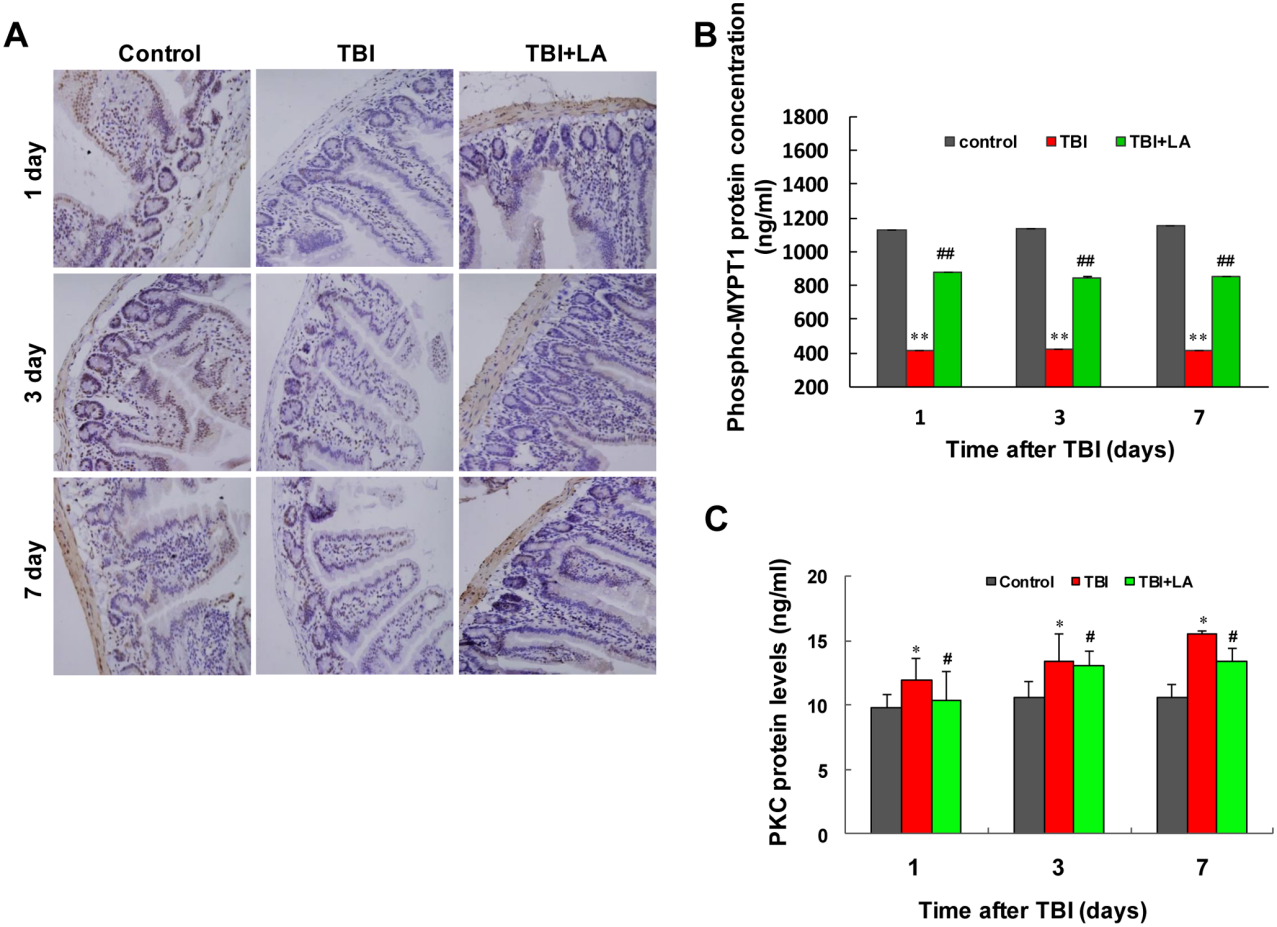
*Lactobacillus acidophilus* attenuated TBI-mediated decrease in levels of phospho-MYPT1 and increases in PKC protein concentrations. The interaction of *Lactobacillus acidophilus* and time on phospho-MYPT1 concentrations was not significant. (A and B) A marked decreases in phospho-MYPT1 were observed in intestinal smooth muscle after TBI, **P < 0.01 compared with control. Treatment with *Lactobacillus acidophilus* significantly attenuated TBI-mediated inhibition of MYPT1 phosphorylation, ##*P* < 0.01 compared with TBI. The interaction of *Lactobacillus acidophilus* and time on PKC protein concentrations was significant. (C) ELISA analysis showed that the concentrations of PKC were increased in intestinal smooth muscle after TBI, *P < 0.05 compared with control. *Lactobacillus acidophilus* significantly attenuated TBI-mediated induction of PKC protein concentrations, #*P* < 0.05 compared with TBI.

It has been shown that MYPT1, a 110–130 kDa MLC-targeting subunit, plays an important role in targeting MLCP to the myosin filaments [27]. Phosphorylation of MYPT1 at Thr853 is thought to decrease PP1c catalytic activity, which inhibits MLCP and maintains MLC phosphorylation [27]. We then examined the effect of *Lactobacillus acidophilus* on MYPT1 in small intestine of TBI mouse model using immunohistochemistry analysis. Table 1 shows that the interaction of *Lactobacillus acidophilus* and time on phospho-MYPT1 was not significant (*P =* 0.36). As shown in Fig 7A and 7B, marked decreases in immunoreactivity for phospho-MYPT1 were observed in intestinal smooth muscle after TBI (*P* < 0.01). Treatment with *Lactobacillus acidophilus* significantly attenuated TBI-mediated inhibition of MYPT1 phosphorylation (*P* < 0.01). Meanwhile, the effect of time on phospho-MYPT1 was significant (*P* < 0.01) (Table 1).

Protein kinase C (PKC) is a serine/threonine kinase known to exist in several isoforms, all which contribute to various physiological activities [28]. It has also been shown that PKC is involved in the regulation of MLC_20_ phosphorylation and has an important role in modulating smooth muscle contractile response [29]. To determine whether PKC is involved in *Lactobacillus acidophilus*-mediated recovery of phosphorylation of MLC_20_ and contraction in intestinal smooth muscle after TBI, we evaluated the effect of *Lactobacillus acidophilus* on PKC protein concentration in small intestine of TBI mouse model using ELISA. Table 1 shows that the interaction of *Lactobacillus acidophilus* and time on PKC protein concentration was not significant (*P* = 0.24). As shown in Fig 7C, marked increases in concentrations of PKC were observed in intestinal smooth muscle after TBI (*P* < 0.05). Treatment with *Lactobacillus acidophilus* significantly attenuated TBI-mediated induction of PKC (*P* < 0.05). Meanwhile, the effect of time on PKC protein concentration was significant (*P* < 0.01) (Table 1). This finding suggests that PKC could play an important role in *Lactobacillus acidophilus*-mediated recovery of phosphorylation of MLC_20_ and contraction in intestinal smooth muscle after TBI.

## Discussion

Traumatic brain injury (TBI) is a recognized public health problem with a reported incidence rate of patients hospitalized for TBI (~22/100 000 or higher) [30]. TBI can lead to several physiologic complications including gastrointestinal dysfunction [31]. It has been shown that TBI caused a significant decrease in intestinal contractile activity, leading to delayed intestinal transit [32]. There was a significant negative correlation of brain volume loss vs contractile activity of small intestine, suggesting a correlation between decreased contractile activity and increased severity of brain injury [32]. Therefore, effectively improving disorder of small intestinal contractile activity is beneficial to improve the clinical outcome of patient after TBI.

Recent evidence revealed that beneficial probiotics show promise for the treatment of disorder of small intestinal contractile activity caused by TBI [33]. *Lactobacillus* is one of the predominant probiotics in the animal’s GI tract which are defined as live microorganisms and when administered in adequate amounts, confer a health benefit to the host [5,34,35]. Maasi M et al. reported that the probiotic preparation VSL#3 (e.g. *Bifidobacterium*, *Lactobacillus*, *Streptococcus*) is able to modulate intestinal motility [13]. Among these probiotic strains, *Lactobacillus* strains stimulated the contraction of ileum segment, whereas all probiotic strains tested induced proximal colon relaxation response [13]. *Lactobacillus acidophilus*, a major component of *Lactobacillus*, exhibits multiple biological functions (e.g. improving intestinal function) [36]. However, the mechanism by which *Lactobacillus acidophilus* improves intestinal function has not been explored. In the present study, we found that the intestinal contractile activity was impaired in mice after TBI, which led to decreased contractile amplitude, frequency, and tension. We also found that *Lactobacillus acidophilus* recovered the intestinal contractile activity in TBI mouse model. Our study demonstrated that *Lactobacillus acidophilus* stimulated the small intestine contraction, which was inhibited by TBI.

Increasing evidence shows that interstitial cells of Cajal (ICC), a pacemaker of gastrointestinal motility, play critical roles in the regulation of gastrointestinal motility [37]. Under certain pathophysiological conditions, loss or defects in ICC networks or numbers are the cause of intestinal motility disorder [38]. In this study, the interruption of ICC networks and reduction of ICC numbers were observed in TBI mouse model. Treatment with *Lactobacillus acidophilus* significantly prevented TBI-mediated disruption of ICC networks and reduction of ICC numbers. Therefore, our findings suggest that the application of probiotics such as *Lactobacillus acidophilus* may significantly improve intestinal contractile activity after TBI through recovery of ICC networks and numbers.

In the present study, we provide detailed molecular mechanistic information how TBI impairs the intestinal contractile activity (i.e. by inhibiting MLCK protein concentration and MLC_20_ phosphorylation, and by inducing MLCP protein concentration). Recent studies have shown that the contractile response to agonists consists of two phases: initial contraction and MLC_20_ phosphorylation mediated by Ca^2+^/calmodulin-dependent activation of MLCK, and sustained contraction and MLC_20_ phosphorylation mediated by a dual pathway involving activation of RhoA, leading to phosphorylation of MYPT1 or CPI-17 by Rho kinase or PKC, respectively. Phosphorylation of both MYPT1 and CPI-17 caused inhibition of MLC phosphatase (MLCP) and resulted in sustained MLC_20_ phosphorylation and contraction [39,40]. Our present results indicate that TBI may simultaneously inhibit initial and sustained contraction and MLC_20_ phosphorylation, and *Lactobacillus acidophilus* significantly prevented TBI-mediated both initial and sustained contraction and MLC_20_ phosphorylation in small intestine of mouse, based on the following evidence: (i) TBI significantly inhibits initial intestinal contraction and MLC_20_ phosphorylation via MLCK inhibition, which are significantly prevented by *Lactobacillus acidophilus*; (ii) TBI markedly inhibits sustained contraction and MLC_20_ phosphorylation through induction of MLCP, which is significantly prevented by *Lactobacillus acidophilus*; (iii) TBI significantly inhibits phosphorylation of MYPT1, a myosin phosphatase targeting subunit, which is prevented by *Lactobacillus acidophilus*. These findings suggest that on one hand, TBI-inhibited MLCK-dependent MLC_20_ phosphorylation and initial intestinal muscle contraction is prevented by *Lactobacillus acidophilus* application. On the other hand, TBI-mediated inhibition of MYPT1/MLCP-dependent MLC_20_ phosphorylation and sustained intestinal muscle contraction is prevented by *Lactobacillus acidophilus*.

In this study, we also found that PKC activity is induced in small intestine after TBI. It has been shown that calmodulin, in the presence of elevated calcium concentration, inhibits PKC and activates MLCK, resulting in phosphorylation of MLC_20_ and contraction [40]. Recent reports showed that inhibition of calcium/calmodulin-dependent kinase II (CaMK II) is necessary for TBI [41], and decreased expression of calmodulin was observed after TBI [42]. Consistence with these reports, our findings suggest that activation of PKC could contribute to inhibition of MLCK, leading to inhibiting phosphorylation of MLC_20_ and intestinal contraction mediated by TBI. However, the exactly mechanism how PKC regulates TBI-mediated inhibition of intestinal contraction through suppression of MLCK-dependent MLC_20_ phosphorylation needs further study to explain.

The absence of observation of food intake was a potential limitation in our study. Evidence suggested that trauma is believed to affect animal food intake [3]. However, no meta-analysis indicated that food intake was associated with the gastrointestinal motility so far. Food consumption was evaluated generally as a nutritional assessment index [43]. Both TBI group and TBI + *Lactobacillus acidophilus* group possess the equal ability of food acquisition in this study due to the same condition of traumatic brain injury. Therefore, we focused on the effects of *Lactobacillus acidophilus* on gastrointestinal motility without considering food intake.

In summary, in this study we showed that TBI markedly suppressed contractile activities of intestinal smooth muscle, which were significantly prevented by probiotic *Lactobacillus acidophilus* application. Mechanistically, we found that TBI inhibited initial intestinal muscle contraction through inhibition of MLCK-dependent MLC_20_ phosphorylation, and these events were prevented by *Lactobacillus acidophilus* application. On the other hand, TBI-mediated inhibition of MYPT1/MLCP-dependent MLC_20_ phosphorylation and sustained intestinal muscle contraction were prevented by *Lactobacillus acidophilus*. These findings may provide a novel mechanistic basis for the application of *Lactobacillus acidophilus* in the treatment of TBI.

## Conclusions

The present study demonstrates that *Lactobacillus acidophilus* efficiently improved the contractile properties of intestinal smooth muscle, which were impaired by TBI. PKC/MLCK/MLC signaling pathway could play an important role in *Lactobacillus acidophilus*-mediated improvement of contractile properties of intestinal smooth muscle after TBI. These findings may provide a novel mechanistic basis for the application of *Lactobacillus acidophilus* in the treatment of TBI.
